# Flavonoid Myricetin Modulates **G** **A** **B** **A**_**A**_ Receptor Activity through Activation of **Ca** ^2+^ Channels and CaMK-II Pathway

**DOI:** 10.1155/2012/758097

**Published:** 2012-11-11

**Authors:** Xiao Hu Zhang, Ze Gang Ma, Dewi Kenneth Rowlands, Yu Lin Gou, Kin Lam Fok, Hau Yan Wong, Mei Kuen Yu, Lai Ling Tsang, Li Mu, Lei Chen, Wing Ho Yung, Yiu Wa Chung, Bei Lin Zhang, Hua Zhao, Hsiao Chang Chan

**Affiliations:** ^1^Epithelial Cell Biology Research Centre, The Chinese University of Hong Kong, Hong Kong; ^2^Department of Physiology, Medical College Qingdao University, Qingdao, China; ^3^Laboratory Animal Services Centre, The Chinese University of Hong Kong, Hong Kong; ^4^Department of Physiology, School of Basic Medical Sciences, Jilin University, Changchun, China; ^5^School of Biomedical Sciences, Faculty of Medicine, The Chinese University of Hong Kong, Hong Kong

## Abstract

The flavonoid myricetin is found in several sedative herbs, for example, the St. John's Wort, but its influence on sedation and its possible mechanism of action are unknown. Using patch-clamp technique on a brain slice preparation, the present study found that myricetin promoted GABAergic activity in the neurons of hypothalamic paraventricular nucleus (PVN) by increasing the decay time and frequency of the inhibitory currents mediated by GABA_A_ receptor. This effect of myricetin was not blocked by the GABA_A_ receptor benzodiazepine- (BZ-) binding site antagonist flumazenil, but by KN-62, a specific inhibitor of the Ca^2+^/calmodulin-stimulated protein kinase II (CaMK-II). Patch clamp and live Ca^2+^ imaging studies found that myricetin could increase Ca^2+^ current and intracellular Ca^2+^ concentration, respectively, via T- and L-type Ca^2+^ channels in rat PVN neurons and hypothalamic primary culture neurons. Immunofluorescence staining showed increased phosphorylation of CaMK-II after myricetin incubation in primary culture of rat hypothalamic neurons, and the myricetin-induced CaMK-II phosphorylation was further confirmed by Western blotting in PC-12 cells. The present results suggest that myricetin enhances GABA_A_ receptor activity via calcium channel/CaMK-II dependent mechanism, which is distinctively different from that of most existing BZ-binding site agonists of GABA_A_ receptor.

## 1. Introduction

*γ*-aminobutyric acid (GABA) is the most important inhibitory neural transmitter in the central nervous system (CNS) and is a key element in maintaining normal physiological brain functions [1]. The fast inhibitory effect of GABA is mediated by GABA_A_, a pentameric ionotropic receptor which is composed of a chloride pore and has numerous regulatory binding sites for barbiturates, benzodiazepines, and neurosteroids [2]. The GABA_A_ receptor is involved in sedation and anxiolysis and has thus become an important pharmacological target for many neurological disorders such as sleep dysfunctions, anxiety, schizophrenia, and epilepsy [3]. It has long been found that increase CNS GABAergic activity will promote sleep, and sedatives such as barbiturates and benzodiazepines are known to bind to their specific binding site on the GABA_A_ receptor as agonists and thus induce sedation [4–6]. Enhancing the GABAergic functions is thus an established major treatment strategy for acute and chronic insomnia. Some GABA_A_ receptor agonists such as Zolpidem and Zopiclone, which are known to interact with the benzodiazepine (BZ) site of GABA_A_ receptor directly, are the most prescribed hypnotics [7, 8]. However, the use of these drugs is not without their problems, reporting a rebound insomnia, next day drowsiness, unrefreshing sleep, or even more serious problems such as tolerance and dependence when being long-term used [9, 10]. Due to the adverse and undesirable side effects of existing sedative drugs and remedies, there is therefore a continuing search for new drugs for insomnia treatment.

Flavonoids are present in almost all dietary and medicinal plants consumed by humans, including tea, fruits, vegetables, wine, and some medicinal herbs. The main biological effects of flavonoids are antioxidant and free-radical scavenging abilities, some also show some vascular and anticancer activities [11]. The effects of flavonoids on the central neural system (CNS) have been revealed decades ago when some flavonoids were found having high affinity to GABA_A_ receptor, resulting in hypnotic, anxiolytic, and anticonvulsive effects in animals, but the results from different tested flavonoids are not consistent and the underlying mechanisms are still not clear [12, 13].

Myricetin (2, 5, 7, 3′, 4′, 5′-pentahydroxylflavonol) is a widely distributed flavonoid [14], which has been reported to have a variety of biological effects, such as anticancer, antioxidant, antidiabetic, prevention of apoptosis, and neuron degeneration [14–17]. Myricetin is also an important bioactive compound present in some medicinal herbs, for example, St. John's Wort [18], which have hypnotic and antidepressant effects. We have recently found that myricetin is one of the main ingredients of *Ampelopsis grossedentata*, which is used as a herbal tea to treat insomnia in China. However, the effects and biological targets of myricetin on the CNS are largely unknown, although its possible action on CNS for its antinociceptive and analgesic effect has been suggested [19–21].

The hypothalamus is a key structure in the brain for sleep generation and regulation and considered as a switch of awake and sleep [22–24]. In the hypothalamus, PVN plays a key role in sleep regulation and has broad interactions with other brain areas including the circadian centre suprachiasmatic nucleus (SCN) and hypocretinergic lateral hypothalamus (LH) [25, 26]. GABA is the main neurotransmitter within PVN, and PVN also has extensive connections with other sleep related transmitters systems, such as hypocretin/orexin, cytokines, and neuropeptide Y [27, 28]. We therefore hypothesize that the hypothalamic PVN may be a CNS target of myricetin for its possible sedative action. 

## 2. Material and Methods

### 2.1. * In Vitro * Brain Slice Preparation

Male Sprague-Dawley rats (18–24 days old) were anaesthetized and decapitated. The brain was immediately removed into ice-cold artificial cerebrospinal fluid (ACSF), consisting of (mM): NaCl 125, KCl 2.0, MgSO_4_ 1.2, CaCl_2_ 2.5, KH_2_PO_4_ 1.2, glucose 11, and NaHCO_3_ 26, and bubbled with 95% O_2_ and 5% CO_2_. 250 *μ*m brain slices containing PVN were sectioned using a vibrating microtome (Integraslice 7550 MM, Camden Instrument). After equilibration for 1 hour at 35°C in ACSF saturated with 95% O_2_ and 5% CO_2_, a slice was transferred to the recording chamber and perfused with ACSF at a rate of 1–1.5 mL/min maintained at a temperature of 34-35°C. 

### 2.2. Patch Clamp Recording

Whole-cell patch clamp recordings from PVN neurons were obtained using a MultiClamp 700A amplifier (Axon Instruments). Electrodes (3–5 MΩ) were positioned on the soma of the PVN neurons. Data were low-pass filtered at 3 kHz, sampled, and saved in hard drive using Compex 8.2 software and Digidata 1322A interface (Axon Instrument) for offline analysis. The internal solution of the electrodes consisted of (mM): KCl 140, EGTA 1.0, MgCl_2_ 2.0, HEPES 10, Na_2_ATP 2.0, Tris GTP 0.4, pH 7.25–7.35. In miniature inhibitory postsynaptic currents (mIPSCs) recording, (±)-2-Amino-5-phosphonopentanoic acid (AP-5, 50 *μ*M) and 6-cyano-7-nitroquinoxaline -2,3-dione (CNQX, 20 *μ*M) were included in the bath solution to eliminate glutamate-mediated synaptic currents. When isolating mIPSCs, tetrodotoxin (TTX, 0.5 *μ*M) was added into the bath solution to eliminate the action potential dependent IPSCs. In Ca^2+^ current recording, the internal solution consisted of (mM): CsCl 135, MgCl_2_ 1.0, HEPES 10, EGTA 10, Na_2_-ATP 4, Tris GTP 0.4, and pH 7.3-7.4. The perfusion solution consisted of: (mM) NaCl 100, TEA-Cl 40, KCl 2.5, BaCl_2_ 5, HEPES 10, glucose 10, 4-AP 5, TTX 1, and pH 7.3-7.4. 

### 2.3. Rat Hypothalamus Neuron Primary Culture and PC-12 Cell Line Culture

Sprague-Dawley rats (4-5 days old) were decapitated and the whole hypothalamus was dissected in Krebs' Ringer solution containing 9 g/100 mL Bovine serum albumin (BSA) and cut into small pieces. Trypsin (2.5 mg/mL) was used to digest the tissue for 8 min at a room temperature. After neutralization with trypsin inhibitor (0.5 mg/mL trypsin inhibitor in Kreb's solution with BSA and 0.1 mg/mL deoxyribonuclease II) for 2 min, the hypothalamic neurons were dissociated with fire-polished Pasteur pipettes. The neurones were spun down and sufficient culture medium added to resuspend the neurons and transfer the suspensions to a coverslip (coated with poly-lysine) in a 35 mm dish with a density of 1 × 10^5^/mL. The neurons were then cultured in Dulbecco's minimum essential medium (D-MEM) supplemented with 10% fetal bovine serum, 100 U/mL penicillin and streptomycin, 25 mM KCl, and 2 mg/mL glucose. The medium was changed every two days and the neurons were cultured for 5 days before use. The proliferation of glia cells were suppressed by Arabinose-C. The PC-12 cell line was ordered from ATCC (CRL-1721). Cells were seeded in four 60 mm dishes with 1 × 10^5^/cm^2^ in D-MEM (supplemented with 10% FBS, 5% horse serum, 100 U/mL penicillin and streptomycin) at 37°C in humidified air containing 5% CO_2_. Culture medium was changed every 2-3 days.

### 2.4. Live Ca^2+^ Imaging

The bicarbonate-buffered Krebs-Henseleit solution (KHS) contained 117 mM NaCl, 25 mM NaHCO3, 4.7 mM KCl, 1.2 mM MgSO4, 1.2 mM KH2 PO4, 2.5 mM CaCl2, and 11 mM D-glucose, pH 7.4, when bubbled with 5% CO_2_, 95% O_2_. The membrane permeable, acetoxymethylester forms of Fura-2 and pluronic F127 were from Molecular Probes (Eugene, OR, USA). Neurons grown on the coverslip were loaded with Fura-2 AM by incubation for 45 mins at 37°C in buffered saline containing 3 *μ*M Fura-2 AM and 1.6 *μ*M pluronic F127. Fluorescence was recorded in an inverted Olympus IX70 microscope equipped with a CCD camera operated with MetaFlour software (Universal Imaging Corp.) was used to obtained images of the fura-2 signal exciting at 340 nm and 380 nm. The ratio of these two signals is directly proportional to the [Ca^2+^]_*i*_.

### 2.5. Immunofluorescence Staining

Hypothalamus neurons cultured on cover-slips were incubated with 0.5% DMSO or 50 *μ*M myricetin in culture medium for 10 min and then fixed by 4% of PFA. After 3 washes by PBS, samples were blocked by 5% normal goat serum for 1 hour, and then incubated with antiphospho-CaMKII (1 : 100, rabbit polyclonal antibody, Cat.3361, Cell Signaling Technology) and antibeta tubulin (1 : 200, mouse monoclonal, Cat.T4026, Sigma) at 4°C overnight. Alexa Fluor 488F (1 : 500, goat antirabbit IgG), and Alexa Fluor 568 (1 : 500, goat antimouse IgG) were used as secondary antibodies. Nuclei were stained by Hoechst 33342.

### 2.6. Western-Blot

0.1% DMSO, myricetin (50 *μ*M), and zolpidem (5 *μ*M) were added in the culture medium of PC-12 cells for 30 min. The cells were harvested in a lysis buffer (RAPI buffer: 50 mM Tris-HCl, pH 8.0, 150 mM NaCl, 1% NP-40, 0.5% Sodium deoxycholate, 0.1% SDS). Total lysates (40 mg per lane) were subjected to SDS-PAGE and were transferred onto nitrocellulose membranes (Schleicher&Schuel, Dasse, Germany). The transferred membrane was blocked by incubating in TBST plus 5% fat-free milk. The membrane was then washed 3 times with TBST and incubated with rabbit antirat CaMK-II (1 : 500) polyclonal antibody, and rabbit antirat phosphor-CaMK-II (1 : 500) polyclonal antibody (Santa Cruz, California, USA) in TBST plus 1.5% fat-free milk at 4°C for overnight. The membrane was subsequently washed with TBST and incubated for 1 hour with peroxidase-conjugated secondary antibody. The membrane was washed 3 times with TBST and then detected by enhanced chemiluminescence (Amersham, Piscataway, NJ, USA).

### 2.7. Statistics

Statistical analysis was performed using Graph Pad Prism Software. Student *t*-test was used to analyze calcium imaging and electrophysiological data. A value of *P* < 0.05 was considered statistically significant. 

## 3. Results and Discussion

### 3.1. Myricetin Prolongs the Half Decay Time of GABA_A_ Receptor-Mediated mIPSCs in PVN Neurons

**Figure 1:**
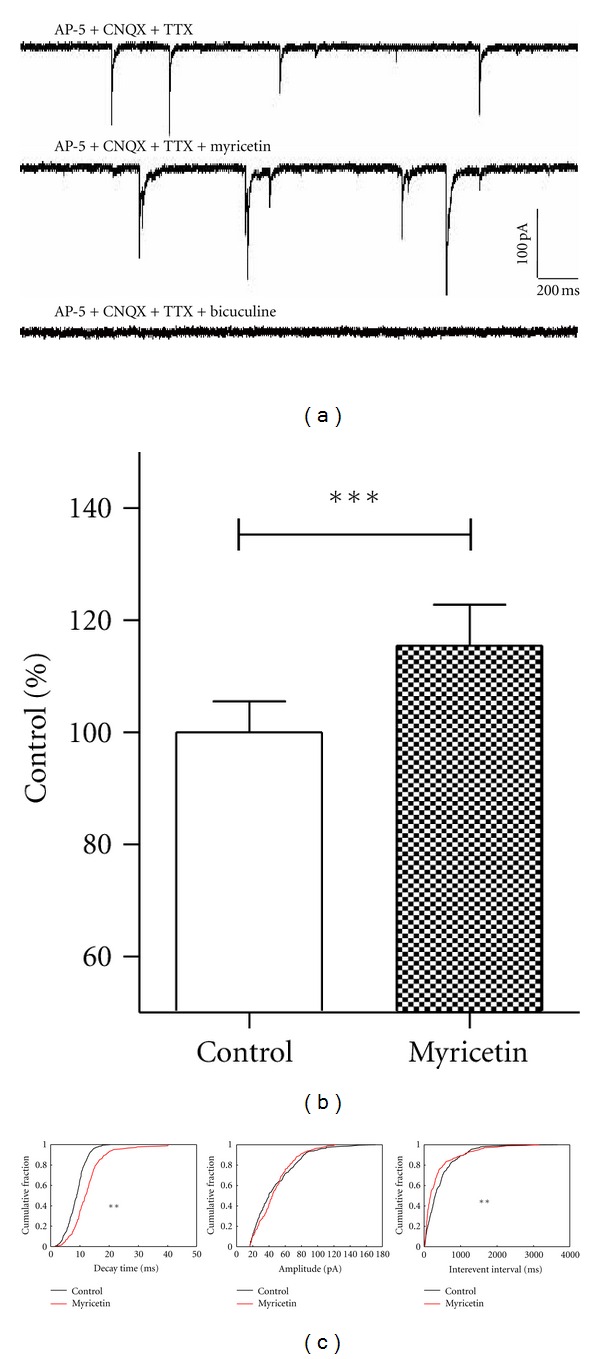
Myricetin increases the half-decay time of GABA_A_ receptor-mediated mIPSCs in PVN neurons. (a) Representative recording of spontaneous GABA_A_ receptor-mediated mIPSCs in PVN neurons from a brain slice preparation at a holding potential of −70 mV with voltage-clamp configuration, and in the presence of AP-5, CNQX, and TTX; (b) bar chart shows myricetin increases the half-decay time of the mIPSCs (*P* < 0.001, paired *t*-test); (c) diagrams show the effects of myricetin on the decay time, amplitude, and the interevent interval of mIPSCs.

We first examined whether myricetin had effect on GABAergic activity of PVN neurons using a brain slice preparation. The spontaneous synaptic currents were recorded at the holding potential of −70 mV. AP-5 (50 *μ*M) and CNQX (20 *μ*M) were used to block glutamate induced excitory post-synaptic currents. TTX (0.5 *μ*M) was used to block the action potential dependent IPSCs. The recorded currents could be totally blocked by GABA_A_ receptor antagonist bicuculine (10 *μ*M), confirming that the currents were the GABA_A_-mediated mIPSCs (Figure 1(a)). In total 14 neurons, myricetin (5 *μ*g/mL) significantly increased the decay time of mIPSCs to 115 ± 7.29% compared with basal (Figures 1(b) and 1(c), *P* < 0.001, paired *t*-test), among which 5 neurons also showed an decreased of the interevent interval, indicating increased frequency of presynaptic GABA release (Figure 1(c), K-S test, *P* < 0.05). These data suggest that myricetin not only modulates the kinetics of post-synaptic GABA_A_ receptors by increasing the decay time, but also influences the spontaneous pre-synaptic GABA release in some cases.

### 3.2. The Action of Myricetin Does Not Involve Direct Interaction with the BZ-Binding Site of GABA_A_ Receptor

**Figure 2:**
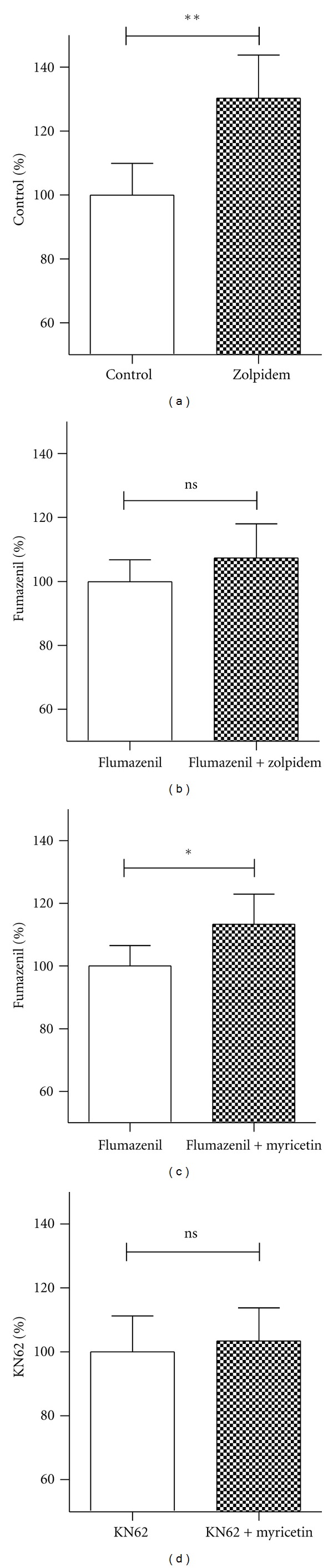
CaMK-II inhibitor KN-62 blocks the effect of myricetin on GABA_A_ receptor in PVN neurons. (a) and (b) Zolpidem increases the half-decay time of mIPSC in the PVN neurons (*n* = 6, *P* < 0.01), and this effect is totally blocked by BZ site antagonist Flumazenil. (c), Flumazenil cannot block the effect of myricetin on the decay time of mIPSC (*n* = 11, *P* < 0.05). (d) The CaMK-II inhibitor KN-62 (15 *μ*M) could completely prevent the increase of decay time induced by myricetin (*n* = 7).

The BZ-binding site is the most important modulating site on the GABA_A_ receptor and most known hypnotics, for example zolpidem, exert their effects by binding to the BZ site of GABA_A_ receptor [29]. To investigate if myricetin also binds to the BZ site, we blocked the BZ site using a specific BZ-binding site antagonist flumazenil. We first tested the effects of Flumazenil on mIPSCs in PVN neurons. Consistent with the previous reports [30], 10 *μ*M flumazenil by itself had no significant effect on the mIPSCs. While 100 nM zolpidem could significantly increase the half decay time (Figure 2(a), 130.3 ± 13.57%; *P* < 0.01), this effect could be totally blocked by preperfusion of 10 *μ*M flumazenil (Figure 2(b), 107.4 ± 10.72%; *P* = 0.32). On the contrary, flumazenil failed to block myricetin-induced increase of decay time of mIPSCs (Figure 2(c), 113.4 ± 9.53%, *P* = 0.042), suggesting that the effect of myricetin on GABA_A_ receptor was not through binding to the BZ site, but rather, through a different mechanism from that of the classic BZ hypnotics such as zolpidem.

### 3.3. Inhibition of CaMK-II Eliminates the Effect of Myricetin on GABA_A_ Receptor in the PVN

Apart from the direct regulatory binding sites on the extracellular domain, the intracellular domains of GABA_A_ receptor can be phosphorylated by multiple protein kinases and thus regulate the channel functions [31]. CaMK-II, which plays an important role in regulating neural excitability, has recently been demonstrated to phosphorylate GABA_A_ receptor subunits in neurons, which in turn not only enhance the inhibitory GABA effects but also influence synaptic plasticity [32–34]. To investigate if CaMK-II was involved in mediating the effect of myricetin, we added 15 *μ*M KN-62, a CaMK-II inhibitor [35], into the internal solution of the patch electrode to inhibit intracellular CaMK-II activity. KN-62 alone had no effects on mIPSCs in PVN neurons, which is consistent with previous reports [36, 37]. After the whole-cell mode was established for 5 min, the mIPSCs were then recorded. In the 7 tested neurons, the myricetin-induced effects on the mIPSCs were abolished (Figure 2(d), 103.5 ± 10.29%, *P* = 0.49), suggesting the involvement of CaMK-II in mediating the effect of myricetin on GABA_A_ receptor.

### 3.4. Myricetin Enhances the Voltage-Dependent Ca^2+^ Channel (VDCC) Currents in the PVN Neurons

**Figure 3:**
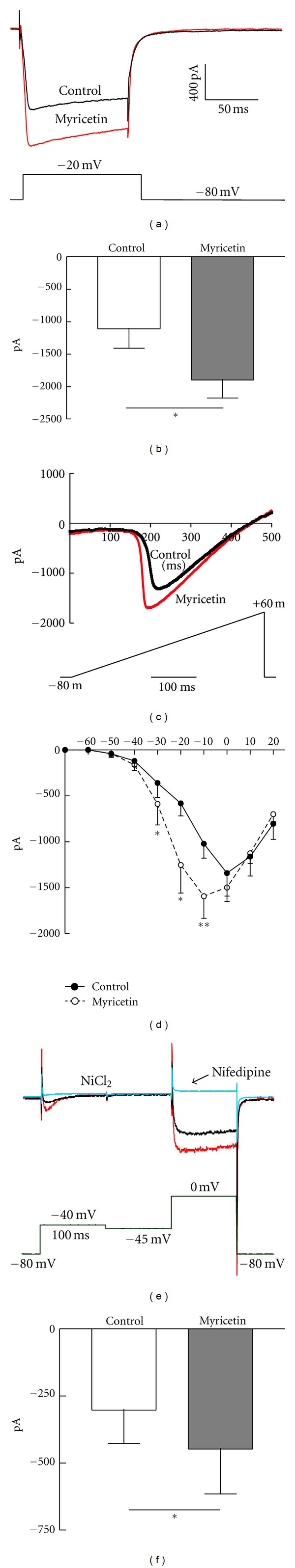
Myricetin activates L-type and T-type Ca^2+^ channels in PVN neurons. (a) Representative recording showing Ca^2+^ current changes in the absence and presence of myricetin (5 *μ*g/mL) in a typical neuron. (b) The statistical data (*t*-test) demonstrate a significant increase of peak current after treatment with myricetin (*n* = 8, *P < 0.05). (c) Effects of myricetin on the Ca^2+^ channel properties in PVN neurons. The current was recorded in response to a 500 ms ramp pulse depolarization from a holding potential of −80 mV to +60 mV, the peak current was increased and shifted to hyperpolarizing direction after myricetin treatment. (d) Summarized current-voltage relationship curves showed the currents amplitude was increased in the region between −30 mV and −10 mV; the peak currents were shifted about 10 mV to negative direction (*n* = 10, *P < 0.05, **P < 0.01). (e) Effects of myricetin on the different types of Ca^2+^ currents in PVN neurons. Representative recording showing the currents changes in the presence (red color) and absence (black color) of myricetin. Myricetin enhanced both T-type and L-type Ca^2+^ currents. The currents were recorded in response to a pulse shown in this figure. The first part of the current can be blocked by 300 *μ*M NiCl_2_ (*n* = 3) and the second part can be blocked by 30 *μ*M nifedipine (*n* = 3) (blue color). (f) The pooled data summarize the effect of myricetin on Ca^2+^ currents when depolarized to −40 mV from a holding potential of −80 mV compared that of control condition (*n* = 8, *P < 0.05).

Intracellular Ca^2+^ increase is the well-known primary trigger of CaMK-II. Previous studies have also demonstrated that myricetin could induce vasoconstriction by activating Ca^2+^ channels in rat vascular smooth muscles [38, 39]. A recent study has also reported that myricetin increases calcium current in rat dorsal root ganglia neurons in a neuropathic pain model [21]. We thus examined the whole Ca^2+^ current of PVN neurons with or without the presence of myricetin.  Figure 3(a) shows typical basal and myricetin induced whole-cell Ca^2+^ currents recorded in PVN neurons by depolarizing the membrane potential from −80 mV to −20 mV in order to active both T-type and L-type Ca^2+^ currents. Myricetin significantly increased the peak Ca^2+^ current by about 50% (Figure 3(b), *P* < 0.05, *n* = 8). The current-voltage relationship was also examined by applying a 500 ms voltage ramp test from −80 mV to +60 mV. Myricetin increased the inward current and shifted the peak current to the hyperpolarizing direction, while had no effect on the reversal potential (Figure 3(c)). The averaged current-voltage curves in response to a 100 ms step pulse from −80 mV to +20 mV with an increment of 10 mV also showed that myricetin significantly enhanced the peak currents in the range between –30 mV and −10 mV and shifted the averaged peak current about 10 mV to hyperpolarizing direction (Figure 3(d)). To separate L-type and T-type Ca^2+^ currents, we depolarized the membrane potential from −80 mV to −40 mV for 100 ms to elicit T-type Ca^2+^ channel current, then followed by a brief hyperpolarizing pulse at −45 mV for 100 ms; and then further depolarized to 0 mV to elicit the L-type Ca^2+^ channel current (Figure 3(e)). The first part of the current was inactivated rapidly, which could be blocked by 300 *μ*M NiCl_2_, consistent with the characteristic of T-type Ca^2+^ channel current. Myricetin significantly increased the T-type Ca^2+^ channel current (Figure 3(f), *P* < 0.05, *n* = 8). The second part of the current was inactivated slowly and blocked by 30 *μ*M nifedipine, confirming high voltage dependent L-type Ca^2+^ current. In 12 cells examined, 4 neurons contained only the high voltage dependent Ca^2+^ currents, other neurons all expressed both L- and T-type Ca^2+^ currents. 

### 3.5. Myricetin Increased Intracellular Ca^2+^ Levels in Rat Hypothalamus Primary Culture Neurons through Both T- and L-type Ca^2+^ Channels. 

**Figure 4:**
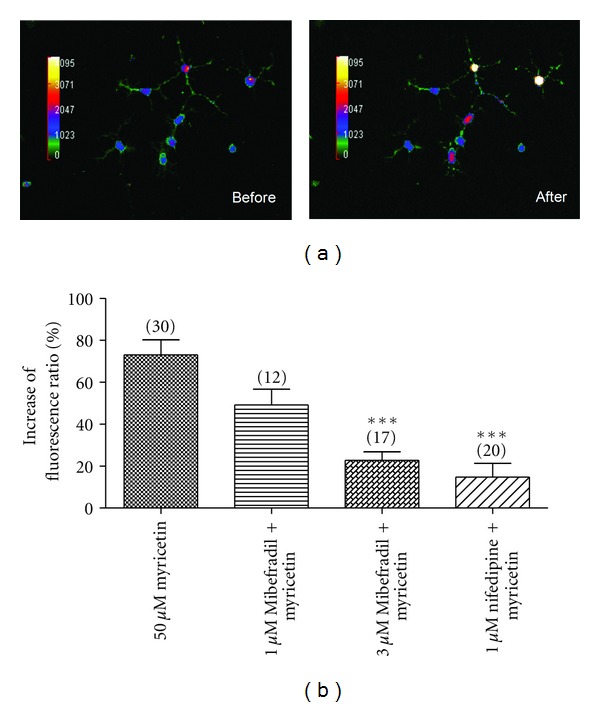
Myricetin-induced intracellular Ca^2+^ increase in the primary culture of rat hypothalamus neurons and the effects of Ca^2+^ channel blockers. (a) Images of rat hypothalamus neurons before and after 50 *μ*M myricetin perfusion, showing increased Ca^2+^ dye intensity; (b) effect of Ca^2+^ channel blockers on myricetin induced intracellular Ca^2+^ increase (***P < 0.001, versus 50 *μ*M myricetin, one-way ANOVA). Mibefradil is T-type Ca^2+^ channel blocker; nifedipine is L-type Ca^2+^ channel blocker. Numbers on the top of each bar represent the numbers of cells examined.

To confirm myricetin activates T- and L-type calcium channels in neurons, we further examined the effect of myricetin on the intracellular Ca^2+^ levels in the hypothalamus primary culture neurons by live Ca^2+^ fluorescent imaging. We found that there were about 50% cells responded to 50 *μ*M myricetin with a fast rise in Fura-2 AM fluorescent intensity, indicating an increase in intracellular Ca^2+^ level (Figure 4(a)). Pretreatment of the cells with different types of Ca^2+^ channel blockers reduced the myricetin-induced Ca^2+^ increases to varied extents, with more potent effects observed with the T-type Ca^2+^ channel blocker, Mibefradil (3 *μ*M), and a L-type Ca^2+^ channel blocker, nifedipine (1 *μ*M), indicating involvement of both T-type and L-type Ca^2+^ channels (Figure 4(b)). 

### 3.6. Myricetin Increased CaMK-II Phosphorylation in Rat Hypothalamus Primary Culture Neurons and PC-12 Cell Line

**Figure 5:**
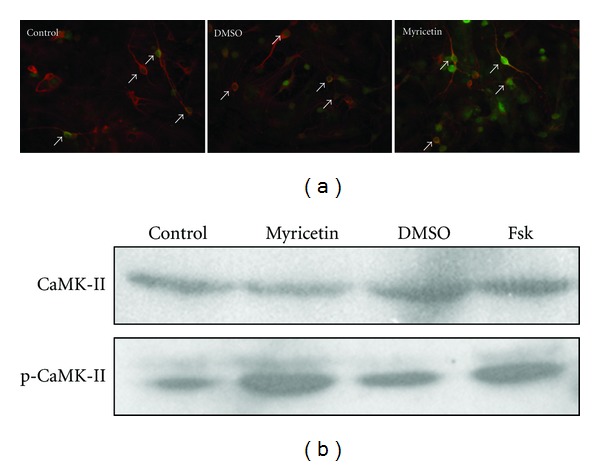
Myricetin increases CaMK-II phosphorylation in primary culture of rat hypothalamic neurons and PC-12 cell line. (a) Myricetin increases CaMK-II phosphorylation in hypothalamus neurons. DMSO: incubated with 0.5% DMSO for 10 min; Myricetin: incubated with 50 *μ*M myricetin for 10 min. Arrows indicate neuronal cell bodies (Green: phosphorylated CaMKII; red: beta-tubulin). (b) Myricetin increases phosphorylated CaMK-II in PC-12 cell line. Control, blank control; DMSO, 0.1% DMSO in PBS; MYR: myricetin, 50 *μ*M in PBS with 0.05% DMSO; FSK: forskolin, 10 *μ*M in PBS with 0.1% DMSO.

The biological activities of CaMK-II are regulated by several phosphorylation sites on its four subunits (*α*, *β*, *γ*, and *δ*). Autophosphorylation of Thr286 in CaMK-II *α* subunit triggered by Ca^2+^ dramatically increases its enzyme activity and affinity to calmodulin, which are essential to many neural process such as synaptic transmissions [40]. We used the primary culture of rat hypothalamus neurons as an *in vitro* model in conjunction with immunofluorescence staining to further investigate if myricetin can indeed increase CaMK-II phosphorylation. We found that after 10 min of incubation with 50 *μ*M myricetin, the phospho-CaMK-II (Thr286) immunoreactivity was significantly increased in the cell body of neurons (Figure 5(a)). Western-blotting result also showed that the phospho-CaMK-II (Thr286) protein was increased after treatment of the neuronal cell line PC-12 with 50 *μ*M myricetin for 30 min, compared with blank and DMSO controls (Figure 5(b), 10 *μ*M forskolin was used as a positive control). Both the immunostaining and western blot results confirmed enhanced CaMK-II phosphorylation by myricetin.

## 4. Conclusions

The present study is the first to demonstrate the modulatory effect of myricetin on GABAergic activity, through a mechanism distinctively different from current GABA_A_ receptor agonists. The present results suggest that myricetin-enhanced GABAergic activity in the hypothalamic PVN neurons is not through direct binding to the regulatory BZ site on GABA_A_ receptors, as is the case with current benzodiazepine sedatives such as zolpidem. The BZ site is the most important allosteric modulatory site of GABA_A_ receptor. Zolpidem is a positive allosteric modulator which binds to the BZ site and increases the efficacy of GABA. Flumazenil acts as a BZ site antagonist, which binds to BZ site and thus blocks zolpidem binding and thus its effect. In the present study, we found that zolpidem and myricetin all increased the decay time of PVN neurons (Figure 1(b) and Figure 2(a)), however, the effect of zolpidem, but not myricetin, was significantly reduced by flumazenil (Figures 2(b) and 2(c)), suggesting that myricetin is acting via a mechanism distinctively different from that of zolpidem.

The present results suggest that the action of myricetin on PVN neurons is through an indirect pathway by first activating T- and/or L-type Ca^2+^ channels on post-synaptic neurons leading to increase in intracellular Ca^2+^level that triggers CaMK-II phosphorylation. Activated CaMK-II has many downstream targets including phosphorylation sites on GABA_A_ receptors, thereby enhancing GABA_A_ channel functions [31]. In the present study, the enhancement of post-synaptic GABAergic activity is indicated by the prolonged decay time of mIPSCs in PVN brain slices (Figure 1). The ability of myricetin to activate Ca^2+^channels, promote intracellular Ca^2+^ increase and influence CaMK-II activity has been consistently demonstrated in the present study on brain slice preparation, primary culture of hypothalamic neurons or PC-12 neuronal cell line. The presently observed ability of myricetin to induce calcium currents is consistent with the recent finding by Hagenacker et al., showing that 10–100 *μ*M myricetin increases calcium current in isolated rat dorsal root ganglia neurons However, the authors also found that at a lower range of concentrations ((0.1–5 *μ*M) myricetin inhibits the calcium current [21], suggesting that different mechanisms of action might be involved. 

While previous studies have demonstrated that myricetin could induce vasoconstriction by activating L-type Ca^2+^ channels in rat vascular smooth muscles [38, 39], the present study is the first to demonstrate that myricetin can also activate T-type Ca^2+^ channels in hypothalamic neurons, which may have important therapeutic implication for insomnia treatment. Activation of Ca^2+^ channels by myricetin in presynaptic neurons may also enhance GABA release as evidenced by the decrease in the inter-event interval, or increase in the frequency of mIPSC (Figure 1(c)). Thus, myricetin may enhance GABAergic activity through both pre-synaptic and post-synaptic mechanisms. 

One of the major problems with existing hypnotics such as zolpidem is that despite their efficacy in promoting sleep initiation, they do not appear to improve sleep quality, with less deep sleep and REM sleep [41, 42]. Fragmented sleep is quite common in insomniacs which results in poor sleep quality and lack of deep sleep and REM sleep [43]. However, the demonstrated ability of myricetin to activate Ca^2+^ channels, particularly T-type Ca^2+^ channels, and to induce changes in intracellular Ca^2+^ levels in hypothalamic neurons may indicate its potential as a novel hypnotic for improving sleep maintenance insomnia. In the thalamus, T-type Ca^2+^ channel activation could block transmission of arousal signals and thus stabilize sleep; deletion of which has been reported to result in fragmented sleep in mice [44]. The pivotal role of T-type Ca^2+^ channel in slow wave sleep generation and maintenance has also been documented [45]. T-type Ca^2+^ channels are activated by small depolarization near the resting membrane potential and therefore regulate the excitability and threshold of neurons. Targeting T-type Ca^2+^ channels as a potential way to improve slow wave sleep in insomniacs has been proposed several years ago, but lacking of T-type Ca^2+^ channel modulators is the main obstacle [46]. As a natural product, the ability of myricetin to activate the T-type Ca^2+^ channel, and modulate GABA_A_ receptor activity makes it a promising new target for investigation. 

The PVN has extend connections with GABAergic, hypocretinergic, cytokines and NPY systems [27, 28], which is also deeply involved in sleep regulation and has broad interactions with other brain areas including the circadian centre suprachiasmatic nucleus (SCN) and hypocretinergic neurons location lateral hypothalamus [25, 26]. The PVN may thus be a potential target area of myricetin in the CNS to exert its potential hypnotic effect. In conclusion, the present study has demonstrated for the first time that myricetin, a flavonol compound found in a number of herbal hypnotics, can enhance GABAergic activity in rat brain slice and neuronal cultures. Whether myricetin can indeed improve sleep quality awaits further investigation *in vivo*, but the GABAergic action of myricetin appears to be distinctively different from that of currently available hypnotics, and its ability to activate Ca^2+^ channels, particularly T-type Ca^2+^ channels, makes it an attractive new candidate for further investigation as a possible sedative agent.
